# NOURISH, Nutritional OUtcomes from a Randomised Investigation of Intradialytic oral nutritional Supplements in patients receiving Haemodialysis: a pilot randomised controlled trial

**DOI:** 10.1186/s40814-015-0007-1

**Published:** 2015-03-29

**Authors:** Louise Jackson, Judith Cohen, Benjamin Sully, Steven Julious

**Affiliations:** 1 Dietetic Department, Sheffield Teaching Hospitals NHS Foundation Trust, Northern General Hospital, Herries Road, Sheffield, S5 7 AU UK; 2 School of Health and Related Research, University of Sheffield, 30 Regent Street, Sheffield, S4 4DA UK

**Keywords:** Nutritional supplements, Haemodialysis, Quality of life, Handgrip

## Abstract

**Background:**

The study was done to assess the feasibility of conducting a trial evaluating the use of an intradialytic oral nutritional supplement (ONS) on nutritional status.

**Methods:**

The study design is a single centre, parallel group, external pilot randomised controlled trial (RCT). The setting was at a haemodialysis unit in Sheffield, UK. The aim was to recruit 30 trial participants to allow at least 12 evaluable patients per arm, but the actual study sample consisted of 10 adults with a body mass index (BMI) ≤22 kg/m^2^, receiving thrice weekly haemodialysis. All participants received nutritional advice from a renal dietitian as per usual practice. The intervention included the provision of an intradialytic ONS. Feasibility outcomes included recruitment to time and retention of participants along with palatability of ONS. Secondary outcomes were clinical parameters to obtain variance and estimates of effect size to inform the sample size calculation for a definitive trial.

**Results:**

Recruitment was undertaken for a fixed period of 6 weeks. Rates were lower than expected mainly due to ineligibility with only 7% of screened patients (19/265) being eligible and 4% (10/265) of these being recruited. Due to the small proportion of patients eligible for the trial, all haemodialysis patients at the specified unit were assessed for eligibility. Data completion rates were low for session questionnaires (23%). Sample sizes derived from variance in secondary outcome measure of handgrip strength and adjusted for a dropout rate of 20% indicate that 189 patients would be required for a definitive RCT, requiring 19 UK haemodialysis units to participate.

**Conclusions:**

A definitive RCT is feasible with some adaptation to exclusion criteria and methodology. The exclusion criteria could be adapted to include an increase in upper limit for BMI. The use of questionnaires at each dialysis session may not be feasible but the inclusion of appetite and supplement consumption data collection at the main assessments would provide similar outcome data. Quality of life assessment using SF-12 would be acceptable.

**Trial registration:**

ISRCTN37431579.

## Background

Malnutrition is reportedly present in 20%–50% of the haemodialysis population [1-4]. It can be described as a nutrient intake lower than the nutritional needs of the individual [1] and contributes to protein and energy wasting (PEW). This has many consequences, including increased mortality and morbidity, decreased quality of life (QOL) and increased risk of hospitalisation [1,4].

Causes of malnutrition in the haemodialysis population are multifactorial but include a decreased appetite and oral intake due to uraemic toxins [5], protein losses on dialysis [6] and the catabolic effect of dialysis [7,8].

Interventions to improve nutritional status and prevent malnutrition, through an increase in nutrient intake, include the following: dietary counselling [9,10], the use of oral nutritional supplements and enteral or parenteral nutrition [11]. These interventions have shown to improve various markers of nutritional status in some way but focus on implementation at home rather than during dialysis, potentially indicating a missed opportunity for nutritional support [12].

UK patients receive on average 3–4 h of haemodialysis, three times per week, and it is this period of time that was explored within this study as a potential means of improving nutritional status by administering an oral nutritional supplement (ONS).

The measurement of nutritional status is difficult and should be classified using a variety of reproducible measures that predict outcome [10]. Previous studies measuring the impact of ONS given to haemodialysis patients have used albumin [13,14], subjective global assessment (SGA) [15,16], QOL [16,17] and anthropometric measures [18]. Handgrip strength (HGS) is also an emerging measure of nutritional status [19,20]. Many of the previous studies recommend the need for further research as the type of nutritional support, the timing of ingestion and which nutritional markers should be used to best assess their efficacy are still not clear. This pilot trial explores some of these parameters and will lead to a more robust definitive trial in the future.

## Methods

The aim of this study was to conduct a randomised controlled external pilot trial of the feasibility of undertaking a study to assess the effect of an intradialytic ONS on the nutritional status of haemodialysis patients [21]. Feasibility outcomes included recruitment to time, retention of participants, barriers to recruitment and palatability of ONS. Secondary outcome measures related to the estimation of a sample size for a definitive trial.

Ethical approval was received after a full review by the National Research Ethics Service Committee. Research governance approval was provided by the sponsoring trust.

Setting: A large adult haemodialysis unit in Sheffield, UK.

Inclusion criteria

- Adult haemodialysis patients (18 years+)
- Received dialysis for at least 6 months
- Haemodialysis at least three times per week at the main haemodialysis unit (not satellite centre)
- BMI ≤22 kg/m^2^

Exclusion criteria

- Amputees
- Patients with significant oedema
- Patients who do not speak fluent English
- Receiving nutritional supplementation prior to study commencing or within 1 month commencement in study
- Known allergies to any ingredients in the ONS
- Those with persistent hyperkalaemia or hyperphosphataemia (defined as the last 3 months)

### Outcomes

The primary outcome was the recruitment rate, calculated as the proportion of those screened and consented to participate in the trial within 6 weeks (a fixed recruitment window). Barriers to recruitment, the impact of excluding non-fluent English speakers and the acceptability of a nutritional intervention study protocol in a UK haemodialysis population were assessed.

Patient preference, palatability of ONS and compliance with the intervention were recorded using a questionnaire completed at each dialysis session and the completion rate of these questionnaires was assessed. QOL was assessed using the SF12-V2® [22] along with the acceptability of using this tool.

Secondary outcomes were clinical endpoints related to the effect of intradialytic ONS on nutritional status: HGS, QOL, weight and dietary intake. Secondary outcomes were measured at baseline, after 1 month and at the end of the trial (2 months after randomisation). These outcomes helped determine the most appropriate outcome measures and timing of data collection points for a larger randomised controlled trial (RCT) and informed the sample size calculation.

### Sample size

This trial was designed to collect data to inform the sample size calculations for a future definitive RCT. The primary outcome was recruitment within a fixed time window of 6 weeks, chosen based on practical considerations such as time and the size of the research team (one data collector) along with estimates of attendance of eligible patients at the recruiting unit. It was estimated that 15 patients per arm would be recruited, a total of 30. A sample size of 12 evaluable patients per arm would give appropriate precision for the sample size estimates [21,23].

### Randomisation and blinding

Participants were randomised to treatment group via a web-based randomisation system provided by a University of Sheffield subsidiary software development company, epiGenesys. This took place at the time of recruitment and was actioned by the main author. The allocated treatment (ONS or standard care) was only disclosed after participants’ details had been recorded and entered in the trial. Randomisation was stratified by gender and age of participant with this stratification list provided by the third author. It was not possible to blind allocation of treatment for patients or clinicians as the manufacture of a placebo ONS would be difficult, and one of the aims of the study was to assess palatability of different ONS to inform how to maximise compliance in a definitive RCT.

All participants received information from a renal dietitian as per standard treatment and continued to attend their usual haemodialysis sessions at a frequency of three times per week.

### Data collection

Logs of patients screened and approached to participate were completed to inform possible recruitment rates for future studies. The time taken to approach patients, request consent and to conduct each assessment was also logged. Anonymised basic details were collected from all potentially eligible patients to allow completion of a CONsolidated Standards Of Reporting Trials (CONSORT) flow chart.

Diet histories were taken using a 48-h recall method, and the accuracy enhanced through the use of a food picture resource [24]. This was then analysed using, “Microdiet”® version 2.8.8, and a daily average of total calories, protein, potassium and phosphate was obtained from the recall.

The HGS was measured in the non-dominant arm with the exception of those with their functioning vascular access in that arm. The participant was in a seated position with the elbow at a 90° angle. HGS was measured three times using a hydraulic hand dynamometer, with no rest period between the tests and then an average calculated.

Routine blood tests along with post dialysis weights were recorded. The SF-12v2® Health Survey [22] was completed by the participants along with the session questionnaires. The session questionnaires assessed the general wellbeing of the participant and the choice of snack eaten on dialysis, and for those allocated to the intervention group, it included the choice of ONS, amount consumed and palatability. The final session questionnaire additionally included some short questions related to the ease of completion of the QOL assessment.

The intervention was the ingestion of one ONS per dialysis session over a 2-month period. The ONS varied in format and nutritional composition but each provided between 200 and 300 kcal and 10.5–12 g protein. Previous studies suggest this level of supplementation would go some way to offsetting the haemodialysis losses [12,25]. Formats included pudding style supplements and milkshake type formulas.

### Statistical analysis

Data from all randomised patients was analysed. Means, ranges and standard deviations were reported for the outcome measures of time taken for assessments, baseline characteristics, handgrip strength, dietary intake, QOL and weight. The trial was not powered to enable statistical comparisons to be performed between groups.

## Results

**Figure 1:**
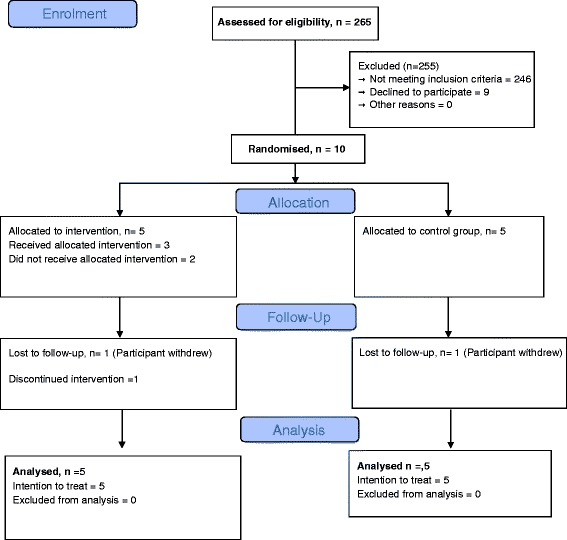
CONSORT flow diagram.

The primary outcome was the proportion of patients screened in a 6-week period that consented and were randomised. Recruitment of *t* = 6 weeks was met but the original target of recruiting 30 participants (12 evaluable patients per arm) was not fulfilled. The CONSORT flow diagram (Figure 1) depicts the numbers of participants screened and eligible.

The whole haemodialysis population within the specified centre was screened for eligibility. In total ten participants were recruited, five to each arm of the trial. This represents a proportion of 4% of patients screened (10/265) that were eligible and consented to participate in the trial.

It is important to highlight that the population we were recruiting from was a prevalent, relative fixed size population and not a presenting population. Prior to the start of the trial, it was anticipated that recruitment and conduct of the trial within the time window would limit the proportion of patients that could be screened. As so few were eligible, 100% of patients were in actuality screened. This demonstrates that participant recruitment cannot be defined by the number of weeks the site is open to recruitment. Further eligible participants would need to be awaited with an extended recruitment time. The eligibility criteria of being on dialysis for at least 6 months is likely to be the only factor to change the number of eligible participants over time.

Only 7.2% (19/265) of the screened population were eligible for recruitment to the trial. The majority of those who were ineligible (71.4%) were excluded due to their BMI being >22 kg/m^2^. The mean BMI of the screened population was 26 kg/m^2^, with a range of 14–52 kg/m^2^. The second greatest reason for exclusion was raised potassium or phosphate level for the previous 3 months (21.9% or 58/265), closely followed by receiving ONS in the previous month or at the time of screening (15.2% or 40/265). The impact of not including non-fluent English speakers excluded 7.2% (19/265) of the screened population. There was no clear indication of future languages that should be included but Urdu, Chinese and Bengali were the main languages at this centre.

Baseline characteristics of the recruited participants can be seen in Table 1. Whilst the age and BMI of participants is similar between the control and intervention groups, it can be seen that time on dialysis is considerably different. This is in part due to the small numbers of participants recruited magnifying the differences in dialysis vintage.

**Table 1:** Baseline characteristics of participants

Characteristic	Allocation	Count	Mean	Standard deviation	Range
BMI in kg/m^2^ at baseline	Treatment	5	19.60	1.09	18–21
	Control	5	20.64	0.84	19.9–22
Age at randomisation	Treatment	5	57	23	26–79
	Control	5	63	20	29–83
Time on dialysis in months	Treatment	5	25	14	6–39
	Control	5	121	125	10–320

The time taken to conduct the assessments can be seen in Table 2. It varied throughout the study with the initial assessment taking longest due to the consent and randomisation procedures.

**Table 2:** Time taken for assessments at key time points

Time of assessment	Count	Mean time taken in minutes	Standard deviation	Range in minutes
Baseline	10	57	16.87	40–90
Month 1	8	35.63	6.23	25–45
Month 2 (study end)	8	38.13	10.33	30–60

The provision of ONS was intended to be after 30 min of being on haemodialysis but prior to significant fluid removal, this was not always achieved but specific data on this was not recorded.

Two patients withdrew from the trial within the first month (20%), one from each arm. The control patient withdrew 2 days after recruitment stating they did not wish “to be bothered” whilst on haemodialysis, the patient withdrew from the treatment arm after 1 month stating that they could not see any benefit to themselves; both consented to the continued use of their baseline data. One additional patient withdrew from the intervention (10%) within the month but allowed data collection to continue at month 1 and study end. The reason given was a dislike of the ONS with some associated gastrointestinal side effects.

### Data completion rates

All participants completed the baseline assessment, and 80% of the control and intervention groups respectively completed assessments at month 1 and study end. Completion rates of the session questionnaires were poor (23.1%) and thus the usefulness of the data is limited.

The completion rates of the QOL tool and its acceptability were encouraging. The SF-12v2® [22] was completed by 100% participants at baseline and 80% at month 1 and study end. Acceptability results indicate that participants did not feel that it took too long to complete and were happy to complete each month.

Results from routine blood tests included biochemical markers used to screen for eligibility and were collected during patient follow-up. Due to the small sample differences between groups, these were not analysed but feasibility aspects were highlighted. Coinciding recruitment of participants with the timing of routine blood tests was not always possible due to the small research team or the tests being conducted outside of the expected window. This had the knock-on effect that results did not always fit with the timing of the other follow-up assessments, differing by up to 10 days on some occasions.

### Outcome measures and sample size for a definitive RCT

The identification of potential outcome measures for a definitive trial and a sample size calculation were crucial elements of this pilot trial. HGS proved easy to use, was accepted by participants and did not result in any adverse events.

Data from the recruited population was used to calculate the required sample size for a definitive trial if the primary outcome were change in HGS. A range of possible minimum clinically important differences were selected from standardised tables of HGS for the general population [26]. See Table 3 for the sample size estimation.

**Table 3:** Sample size estimation based on changes in handgrip strength

Minimum clinically important difference (kg)	80% power	85% power	90% power	95% power
2	450	515	603	745
4	113	129	151	187
6	50	58	67	83
10	18	21	25	30

Type 1 error of 0.05 and standard deviation of 10.705 (calculated from this pilot sample). Increase by 20% to account for dropout rate.

One hundred eighty-nine participants would be required, with 90% power and a 4-kg minimal clinically important difference. This would allow appropriate statistical analysis of the sample whilst the 4 kg was chosen as a significant change in HGS within a relatively short space of time, for example 3 months. The sample size is based on the estimation of 151 participants required as shown in Table 3 and inflating by 20% to account for the dropout rate seen in this pilot trial. However, a further inflation of 10% would be required if a per protocol analysis was to be conducted as a primary analysis. Based on these figures and results from this pilot trial, 19 centres would be required to participate, each with a population of approximately 250 haemodialysis patients recruiting 10 participants each.

## Discussion

The outcomes of this pilot trial focused on feasibility elements, including recruitment to time, eligibility criteria and sample size estimations with the overall aim of assessing if it is possible to conduct a definitive trial within this population.

### Recruitment

The target of recruit to time was met but this did not translate to the expected number of eligible patients being recruited. Eligibility of the screened population was much lower than expected, indicating that the inclusion criteria may have been too stringent.

The exclusion criteria of BMI ≤22 kg/m^2^ was based on published evidence that a BMI at the lower end of the normal range can increase mortality in the haemodialysis population [27]. However, body composition is thought to play a much greater role in the protective effects of a greater BMI, than the BMI itself [28]. The use of BMI as a screening tool was a quick and easy measure but the level of ≤22 kg/m^2^ should be reassessed prior to a definitive trial. If the BMI was raised to ≤24 kg/m^2^ then this would have increased potential recruitment by 10%.

The addition of inclusion criteria such as weight loss over a specified period of time, HGS or additional anthropometric measures should be considered to enhance the detection of participants most at risk of malnutrition.

The exclusion of non-English speakers did contribute to recruitment issues, but no particular language was identified to be beneficial to include in future studies.

The exclusion of those with hyperphosphataemia or hyperkalaemia was a safety precaution for this pilot trial due to the limited funding and monitoring capabilities. It may be possible in a larger trial to include these participants and monitor the effect that the standard ONS has on their already elevated potassium and phosphate levels. The use of a renal-specific ONS, containing reduced electrolytes could also be considered for these participants. If these participants had not been excluded, an additional 53 patients could potentially have been eligible for the trial—although they may still be ineligible due to other exclusion criteria.

We would recommend the use of a web-based randomisation system as it was very easy to use, ensured a reduction of allocation bias and provided an audit trail of the process, essential for the validity of clinical trials [29,30]. However, the use of a wireless device should be considered to potentially reduce the time taken to log into the system in a dialysis centre setting.

The timing of the intervention was considered in the trial. The ONS should have been provided after 30 min of commencing dialysis and before significant fluid removal had commenced with the aim of reducing potential blood pressure-related side effects [31]. In practice, ONS were not always provided within the stated time points, but the exact timing of the supplement was not recorded. Two patients reported detrimental effects during informal discussions having received the ONS towards the end of their dialysis session and felt sick and/or vomited which had caused them to stop the intervention. There is insufficient evidence to suggest that the timing of the ONS was the sole cause of this. However, the use of a prescriptive time period along with a formal log of the time the ONS was provided would have been beneficial and is recommended for future studies. The small sample size prevents definitive timings from being suggested.

The logistics of matching the monthly blood tests to the time of recruitment proved difficult, but in a larger trial, the effect of the blood tests not matching time of recruitment would be lessened as recruitment time could be adapted, and with a longer intervention period, the precise date of the blood tests would be less of a problem as an average effect could still be elicited.

### Data collection

Completion rates of the session questionnaires were very poor (23.1% completed). During informal discussions, participants reported to frequently forget to complete them making monitoring of compliance with the intervention difficult to conclude. However, the three participants in the intervention arm that did not actively stop taking the ONS reported to not take an ONS every dialysis session as indicated. Reasons for this included forgetting to ask for an ONS; not being given a supplement and not feeling like having a supplement at that particular dialysis session. The average consumption over time should potentially be assessed rather than individual session consumption and could be an additional measure asked at the specified time points. In addition to this, the palatability of the ONS could also be assessed on an average basis. There was no indication within this pilot to suggest a certain dislike for particular products although it is well known that ONS preferences vary amongst individuals and the prevention of taste fatigue with variety is very important.

It is essential that dietary intake is monitored to detect any changes to intake that may occur as a result of the ONS. The omission of this monitoring in any definitive trial foregoes the possibility of detecting change solely due to the ONS.

The use of 48-h recall as the method of dietary assessment caused some problems with data capture. Many patients were unable to recall a full 48-h period and therefore the 48-h recall was a patchwork of meals providing an average intake. It provided an estimation of their nutritional intake at the time of the assessment, but the accuracy would be questionable. This was in addition to the well-known problems of dietary assessment including over- and underestimation of intake and the tendency for participants to inform the investigator of food intake that they feel is correct rather than their actual intake [32]. Consideration could be given to the use of an application to record food intake for 2 days either via a website or on a smart phone. Patients could then be given the choice of this application, recall with a trial dietitian or completion of a food diary. The use of an application or diary would still need verification by a research team member to accurately assess portion sizes and to clarify ambiguous entries.

The process of dietary assessment requires significant skill that is part of a dietitian’s training, so if this task was to be delegated to another individual within any future research teams, it would need careful consideration. The standardisation of the process is difficult as individuals respond to questions differently and assessments must not be leading, whilst ensuring that sufficient detail is gathered to allow dietary analysis to be conducted.

The use of the picture resource [24] facilitated portion sizes to be clarified with the patient and improved the accuracy of the assessment along with the ease of inputting the data into the dietary analysis package.

All analysis was performed on an intention to treat (ITT) basis due to the small sample size. A per protocol analysis in addition to the ITT would be indicated in a larger trial.

The SF12-v2® [22] was well received by participants, but there are however, a few considerations for the future use of this tool. Some patients needed help to complete the form due to a number of reasons including inability to use a pen whilst on dialysis due to their vascular access, forgetting reading glasses and also requiring help to understand the questions. The use of both a self-completion and a scripted version of the questionnaire would have prevented these problems.

The changes in weight, target weight and BMI seen in this pilot are very small due to the short data collection period, and their significance cannot be commented upon due to the small sample size. However, in a longer definitive trial, they would be useful tools along with biochemical markers to determine change in nutritional status [1].

### Sample size calculation

The data collected in this pilot trial provided an estimate to calculate sample sizes needed for a definitive trial. There was insufficient data to assess true variation and thus only estimates are provided. These sample size calculations are given in Table 3. HGS was used to calculate the sample size as this is the most likely outcome measure for a definitive trial. It would allow an assessment of body composition change as opposed to weight which can be affected by other parameters. This change in body composition is an important factor in nutritional status and the protective effect of having a greater BMI [28,33].

The change of 2–10 kg in handgrip strength is the average, but it is difficult to state a significant change as there are no standard tables for use in the renal population. Previous studies have shown correlation between HGS and other markers of nutritional status [19,20] and more recently a link with mortality [34], demonstrating the potential effectiveness of this outcome measure. However, it is acknowledged that currently in the haemodialysis population the use of HGS focuses on changes within the individual and further validation of HGS with the production of standard tables for this population would be advantageous prior to conducting a definitive trial. Based on previous research, we have presented possible sample sizes on a range of minimal clinically important differences which should be useful when planning a definitive trial.

### Feasibility of a definitive trial

The trial would be feasible with these estimates as there are over 70 dialysis units in the UK. However, a reduction of centres from 19 would seem more feasible and manageable for a definitive trial. Simple, stratified randomisation was used in this pilot which allowed feasibility outcomes to be assessed. However, consideration could be given to the randomised withdrawal [35] or a “1 month on” and “1 month off” method in a larger trial. This would provide better outcomes regarding the length of time an intervention is required to produce a beneficial effect. For example, an ONS could be provided for a decreasing number of months in different arms of the trial and difference in outcomes assessed or could be provided for 1 month with an alternating month of no ONS and again compare the results. This could potentially reduce costs of providing such an intervention in the longer term and also prevent taste fatigue of the ONS. If the same benefit is seen after 2 months of supplementation as after 6 months, then ONS could be given for shorter periods of time.

The integration of a nested, internal pilot in the definitive trial should also be considered to allow continued monitoring of the feasibility, in particular, the assessment of using different inclusion criteria and the recommended changes to the data collection methods, particularly within the first year of recruitment. The use of a qualitative element to assess the participants’ views on data collection methods would also be beneficial.

The small sample size of this trial is a limitation, but the results provide invaluable information regarding feasibility of conducting a definitive trial of intradialytic ONS in a UK haemodialysis population.

## Conclusions

This study reports on the feasibility of conducting a randomised trial of intradialytic ONS to assess the effect on nutritional status. A definitive trial powered to detect a change in handgrip strength would be feasible in the UK population, with some adaptations to the protocol. The study discusses aspects of the trial that would need further consideration including the eligibility criteria, adherence to the intervention and the choice of outcome measures and assessments.

## References

[1] Fouque D, Kalantar-Zadeh K, Kopple J, Cano N, Chauveau P, Cuppari L (2008). A proposed nomenclature and diagnostic criteria for protein-energy wasting in acute and chronic kidney disease. Kidney Int.

[2] Aparicio M, Chauveau P, Azar R, Canaud B, Laville M, Leverve X (1999). Nutritional status of haemodialysis patients : a French national cooperative study. Nephrol Dial Transplant.

[3] Kopple JD (1997). Protein-energy malnutrition in maintenance dialysis patients. Am J Clin Nutr.

[4] Marcen R, Teruel J, Angel De La Cal M, Gamez C (1997). The impact of malnutrition in morbidity and mortality in stable haemodialysis patients. Nephrol Dial Transplant.

[5] Carrero JJ, Aguilera A, Stenvinkel P, Gil F, Selgas R, Lindholm B (2008). Appetite disorders in uremia. J Ren Nutr.

[6] Wolfson M, Jones MR, Kopple JD (1982). Amino acid losses during hemodialysis with infusion of amino acids and glucose. Kidney Int.

[7] Ikizler TA (2007). Protein and energy intake in advanced chronic kidney disease: how much is too much?. Semin Dial.

[8] Kalantar-Zadeh K, Ikizler TAA, Block G, Avram MM, Kopple JD (2003). Malnutrition-inflammation complex syndrome in dialysis patients: causes and consequences. Am J Kidney Dis.

[9] Akpele L, Bailey JL (2004). Nutrition counseling impacts serum albumin levels. J Ren Nutr.

[10] Fouque D, Vennegoor M, ter Wee P, Wanner C, Basci A, Canaud B (2007). EBPG guideline on nutrition. Nephrol Dial Transplant.

[11] Stratton RJ, Bircher G, Fouque D, Stenvinkel P, de Mutsert R, Engfer M (2005). Multinutrient oral supplements and tube feeding in maintenance dialysis: a systematic review and meta-analysis. Am J Kidney Dis.

[12] Kalantar-Zadeh K, Ikizler TA (2013). Let them eat during dialysis: an overlooked opportunity to improve outcomes in maintenance hemodialysis patients. J Ren Nutr.

[13] Sharma M, Rao M, Jacob S, Jacob CK (2002). A controlled trial of intermittent enteral nutrient supplementation in maintenance hemodialysis patients. J Ren Nutr.

[14] Kalantar-Zadeh K, Braglia A, Chow J, Kwon O, Kuwae N, Colman S (2005). An anti-inflammatory and antioxidant nutritional supplement for hypoalbuminemic hemodialysis patients: a pilot/feasibility study. J Ren Nutr.

[15] Fouque D, McKenzie J, de Mutsert RR, Azar R, Teta D, Plauth M (2008). Use of a renal-specific oral supplement by haemodialysis patients with low protein intake does not increase the need for phosphate binders and may prevent a decline in nutritional status and quality of life. Nephrol Dial Transplant.

[16] Calegari A, Barros EG, Veronese FV, Thome FS (2011). Malnourished patients on hemodialysis improve after receiving a nutritional intervention. J Bras Nefrol.

[17] Scott MK, Shah NA, Vilay AM, Thomas J, Kraus MA, Mueller BA (2009). Effects of peridialytic oral supplements on nutritional status and quality of life in chronic hemodialysis patients. J Ren Nutr.

[18] Beutler KT, Park GK, Wilkowski MJ (1997). Effect of oral supplementation on nutrition indicators in hemodialysis patients. J Ren Nutr.

[19] Leal VO, Mafra D, Fouque D, Anjos LA (2011). Use of handgrip strength in the assessment of the muscle function of chronic kidney disease patients on dialysis: a systematic review. Nephrol Dial Transplant.

[20] Silva LF, Matos CM, Lopes GB, Martins MTS, Martins MS, Arias LU (2011). Handgrip strength as a simple indicator of possible malnutrition and inflammation in men and women on maintenance hemodialysis. J Ren Nutr.

[21] Jackson L, Sully B, Cohen J, Julious S (2013). Nutritional outcomes from a randomised investigation of intradialytic oral nutritional supplements in patients receiving haemodialysis, (NOURISH): a protocol for a pilot randomised controlled trial. Springerplus.

[22] Mark M (2012). User’s Manual for the SF-12v2 Health Survey.

[23] Julious SA (2005). Sample size of 12 per group rule of thumb for a pilot study. Pharm Stat.

[24] Cheyette C, Balolia Y (2013). Carbs & Cals.

[25] Pupim LB, Majchrzak KM, Flakoll PJ, Ikizler TA (2006). Intradialytic oral nutrition improves protein homeostasis in chronic hemodialysis patients with deranged nutritional status. J Am Soc Nephrol.

[26] Bohannon RW, Peolsson A, Massy-Westropp N, Desrosiers J, Bear-Lehman J (2006). Reference values for adult grip strength measured with a Jamar dynamometer: a descriptive meta-analysis. Physiotherapy.

[27] Kopple JD, Zhu X, Lew NL, Lowrie EG (1999). Body weight-for-height relationships predict mortality in maintenance hemodialysis patients. Kidney Int.

[28] Kalantar-Zadeh K, Streja E, Kovesdy CP, Oreopoulos A, Noori N, Jing J (2010). The obesity paradox and mortality associated with surrogates of body size and muscle mass in patients receiving hemodialysis. Mayo Clin Proc.

[29] Schulz KF, Altman DG, Moher D (2010). CONSORT, Statement: updated guidelines for reporting parallel group randomised trials. BMJ.

[30] Viera AJ, Bangdiwala SI (2007). Eliminating bias in randomized controlled trials: importance of allocation concealment and masking. Fam Med.

[31] Strong J, Burgett M, Buss LM, Carver M, Kwankin S, Walker D (2001). Effects of calorie and fluid intake on adverse events during hemodialysis. J Ren Nutr.

[32] Beaton G, Burema J, Ritenbaugh C (1997). Errors in the interpretation of dietary assessments. Am J Clin Nutr.

[33] Leal VO, Moraes C, Stockler-Pinto MB, Lobo JC, Farage NE, Velarde LG (2012). Is a body mass index of 23 kg/m^2^ a reliable marker of protein-energy wasting in hemodialysis patients?. Nutrition.

[34] Matos CM, Silva LF, Santana LD, Santos LS, Protásio BM, Rocha MT (2014). Handgrip strength at baseline and mortality risk in a cohort of women and men on hemodialysis: a 4-year study. J Ren Nutr.

[35] Mallinckrodt C, Chuang-stein C, Mcsorley P, Schwartz J, Archibald DG, Perahia DG (2007). A case study comparing a randomized withdrawal trial and a double-blind long- term trial for assessing the long-term efficacy of an antidepressant. Pharm Stat.

